# Reflexive eye movement alterations are related to subclinical impulsive-compulsive behaviors in Parkinson’s disease

**DOI:** 10.1007/s10072-026-09177-z

**Published:** 2026-06-19

**Authors:** Lenka Hapakova, Igor Straka, Jan Necpal, Alice Kusnirova, Pavol Martis, Peter Valkovic, Zuzana Kosutzka

**Affiliations:** 1 2nd Department of Neurology, Comenius University Faculty of Medicine, University Hospital Bratislava, Limbova 5, Bratislava, 833 05 Slovakia; 2 Neurology Department, Hospital Zvolen, a. s., Kuzmanyho nabrezie 28, 960 01 Zvolen, Slovakia; 3 Centre of Experimental Medicine of the Slovak Academy of Sciences, Institute of Normal and Pathological Physiology, Dubravska cesta 9, Bratislava, 841 04 Slovak Republic

**Keywords:** Parkinson’s disease, Subclinical impulsive–compulsive behaviors, Express saccades, Levodopa equivalent daily dose, Quality of life

## Abstract

**Introduction:**

Subclinical impulsive–compulsive behaviors (s-ICBs) in Parkinson’s disease (PD) are common, clinically relevant, and frequently underdiagnosed. Objective behavioral measures reflecting vulnerability to impulsive–compulsive behavior are lacking. We investigated whether oculomotor measures of inhibitory control are associated with s-ICBs in dopaminergically treated PD patients without clinically manifest impulse control disorders.

**Methods:**

Twenty-nine patients with PD (Hoehn and Yahr stages 1–2) and twenty age-matched healthy controls completed prosaccade and antisaccade eye-tracking tasks. Executive functioning, trait impulsivity, quality of life and s-ICBs were assessed. Group comparisons and association analyses were performed using nonparametric methods with false discovery rate correction. Exploratory mediation analysis examined relationships between levodopa equivalent daily dose (LEDD), s-ICBs severity, and express prosaccades. All assessments were performed in the ON-medication state.

**Results:**

Compared with healthy controls, patients with PD showed a higher frequency of express prosaccades and prolonged antisaccade latencies. Within the PD group, express prosaccades were moderately to strongly associated with s-ICBs severity and LEDD and were also related to reduced quality of life. Mediation analysis revealed a significant statistical indirect effect of dopaminergic medication on express prosaccades through s-ICBs severity, indicating that behavioral symptoms statistically accounted for a substantial proportion of this association.

**Conclusions:**

These findings demonstrate a close relationship between oculomotor control, subclinical impulsive–compulsive behaviors, and dopaminergic treatment in PD. The results highlight the complexity of non-motor manifestations in PD and underscore the need for longitudinal and mechanistic studies to clarify their clinical significance.

**Supplementary Information:**

The online version contains supplementary material available at 10.1007/s10072-026-09177-z.

## Introduction

Impulsive–compulsive behaviors (ICBs) are among the most disruptive non-motor complications of Parkinson’s disease (PD), with substantial consequences for patients, caregivers, and treatment adherence. They comprise repetitive behaviors characterized by impaired self-control and persistence despite adverse consequences, including pathological gambling, compulsive buying, hypersexuality, binge eating, and related behaviors such as punding and hobbyism. Dopaminergic replacement therapy (DRT), particularly dopamine agonists, is a well-established risk factor for clinically manifest impulse control disorders (ICDs) in PD, highlighting the central role of dopaminergic treatment in the emergence of ICBs [1].

While the clinical burden of manifest ICDs in PD is well recognized, impulsive–compulsive symptoms that do not meet diagnostic criteria for ICDs, i.e. subclinical impulsive–compulsive behaviors (s-ICBs), remain under-investigated, despite growing evidence of their clinical relevance. In both research and clinical contexts, s-ICBs refer to the presence of impulsive–compulsive symptoms below the threshold for an ICD diagnosis and are commonly operationalized using the Questionnaire for Impulsive–Compulsive Disorders in Parkinson’s Disease–Rating Scale (QUIP-RS), with scores ≥ 1 indicating symptom presence and higher scores reflecting greater symptom severity [2].

Epidemiological studies show that s-ICBs are common, detectable early in the disease course, and associated with reduced patient- and caregiver-rated quality of life. Importantly, the majority of s-ICBs remain clinically unrecognized. In de novo and drug-naïve PD cohorts, clinically manifest ICDs are reported in approximately 2–3% of patients, whereas ~ 15–20% exhibit s-ICBs on screening instruments such as the QUIP-RS [3, 4]. Despite their prevalence and impact, s-ICBs lack objective behavioral correlates that could support early identification or routine monitoring.

ICBs in PD are linked to a stable tendency toward impulsivity. Trait impulsivity, as measured by the Barratt Impulsiveness Scale (BIS-11), increases gradually from healthy controls (HC) to PD patients without ICDs and further to those with ICDs [5]. Executive dysfunction is also implicated, with poorer performance on tasks assessing cognitive flexibility and set-shifting reported particularly in PD patients with ICDs [6].

Translating ICBs into objective behavioral markers remains challenging and is often examined using inhibitory control paradigms that assess suppression of automatic or inappropriate responses [7]. However, performance-based tasks frequently engage distinct inhibitory subprocesses and often show weak associations with self-report measures, limiting their ecological validity [8]. This underscores the need for theoretically grounded frameworks that disentangle specific inhibitory mechanisms relevant to impulsive behavior. Within contemporary computational models of inhibitory control, impulsivity is conceptualized as dysregulation of fast, reflexive inhibitory processes mediated by the hyperdirect cortico–subthalamic pathway, rather than solely deficits in slower, voluntary control supported by striatal circuits. Both mechanisms are altered in PD [9, 10], but their relative contribution to subclinical impulsive–compulsive behavior remains unclear.

Eye-tracking paradigms provide a sensitive method for dissociating the two inhibitory mechanisms: prosaccade (PS) tasks index automatic orienting responses, whereas antisaccade (AS) tasks assess voluntary inhibitory control. Express prosaccades (EP; 90–140 ms) reflect rapid, stimulus-driven oculomotor responses generated with minimal top-down control, while AS latencies (> 140 ms) and directional errors reflect failures of voluntary inhibition [11]. If s-ICBs reflect subtle impairments in rapid inhibitory gating, they should be detectable using measures sensitive to reflexive oculomotor disinhibition, such as EP.

Previous eye-tracking studies demonstrate systematic alterations in oculomotor control in PD, reflecting basal ganglia dysfunction and dopaminergic modulation. Compared with HC, PD patients show prolonged AS latencies and an increased frequency of EP [9, 12]. DRT can improve voluntary AS performance [13] while, in susceptible individuals, facilitating rapid, stimulus-driven responses, including EP [14, 15].

While several studies have linked impaired AS performance to clinically manifest ICDs in PD [16, 17], no study has examined whether oculomotor measures are associated with s-ICBs treated as a continuous phenotype. Moreover, trait impulsivity and oculomotor indices have not been concurrently examined in PD. This represents a critical gap, given the high prevalence and clinical relevance of s-ICBs.

The present study aimed to examine whether oculomotor measures of reflexive and voluntary inhibitory control are associated with the severity of s-ICBs in dopaminergically treated patients with PD. Secondary objectives were to characterize associations with dopaminergic medication dose, trait impulsivity, global executive functioning, and quality of life, and to contextualize these findings by comparison with HC.

Specifically, we asked:

(i) Do PD patients exhibit alterations in reflexive and voluntary oculomotor inhibitory control compared with HC?
(ii) Within patients with PD, are reflexive and/or voluntary oculomotor measures associated with s-ICBs severity, and do they show distinct patterns of association with s-ICBs severity, dopaminergic medication dose, trait impulsivity, global executive functioning, and quality of life?

## Materials and methods

### Subjects

Patients were recruited consecutively from the Second Department of Neurology at University Hospital Bratislava between April 2022 and March 2024. HC were recruited through public advertisements during the same period. The project was approved by the Ethics Committee of University Hospital Bratislava, with Institutional Review Board approval obtained on March 17, 2022 (identification number 10/2022). Written informed consent was obtained from all participants prior to study procedures.

The patient inclusion criteria for participation in the study were idiopathic PD with mild to moderate disease severity (Hoehn and Yahr stages 1–2), diagnosed according to Movement Disorder Society criteria. Patients were receiving DRT, including levodopa and/or dopamine agonists, on a stable medication regimen for at least one month prior to assessment. The levodopa equivalent daily dose (LEDD) was calculated using a standard conversion protocol [18]. Although dopamine agonists are a known risk factor for ICDs, the present study was not powered to detect medication-class–specific effects; therefore, LEDD was used as the primary dopaminergic exposure variable, with exploratory analyses examining dopamine agonist use separately. Participants had no comorbid conditions expected to interfere with task performance, as detailed below.

The main exclusion criterion for both PD and HC was the presence of ICDs. The following ICDs were considered: pathological gambling, hypersexual disorder, binge eating, and compulsive buying. ICDs were assessed using DSM-5-TR–informed clinical evaluation conducted by a neurologist specializing in movement disorders, incorporating heteroanamnesis obtained from the patient’s caregiver when available. Clinically significant ICDs were defined as ICBs associated with marked adverse consequences, including financial debt, strained interpersonal relationships, social withdrawal, or health-related harm. In line with prior work [19], ICD diagnosis was based exclusively on clinical assessment. In PD group, QUIP-RS scores, treated as continuous spectrum, were analysed to quantify s-ICBs burden [20]. To minimize misclassification, ICD diagnosis was based on structured clinical evaluation rather than questionnaire thresholds alone. Patients with behavioral symptoms causing functional impairment were excluded, even if QUIP-RS scores were modest. HC also completed the QUIP-RS as an additional screening measure, with eligibility requiring domain scores < 2 across all ICD-related and related behavior domains, consistent with prior validation studies [1, 19]. The dopamine dysregulation syndrome domain was omitted from HC assessment.

Additional exclusion criteria for both groups included clinically significant depression or dementia, other neurological or psychiatric disorders, use of medications known to affect eye movements (e.g., benzodiazepines), and ocular or visual system disorders that could impair visual acuity.

### Examination protocol

**Fig. 1:**
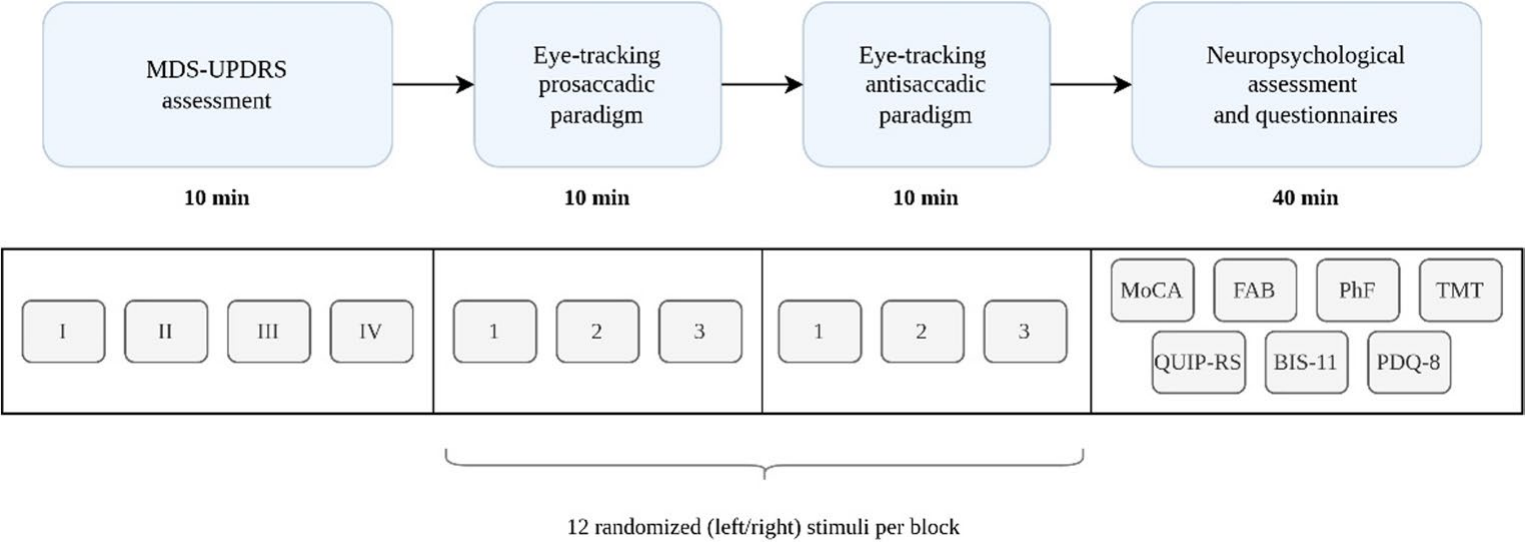
Examination protocol. The fixed sequence of the examination protocol, comprising clinical motor assessment, eye-tracking tasks (prosaccade and antisaccade), neuropsychological testing, and questionnaire-based evaluations, with approximate durations indicated. BIS-11 = Barratt Impulsiveness Scale; FAB = Frontal Assessment Battery; MoCA = Montreal Cognitive Assessment; PDQ-8 = Parkinson’s Disease Questionnaire–8; PhF = phonemic verbal fluency; QUIP-RS = Questionnaire for Impulsive–Compulsive Disorders in Parkinson’s Disease–Rating Scale; TMT = Trail Making Test. The schematic was created using draw.io

Assessments were conducted in the optimal ON-medication state, approximately 1 h after the morning dose of antiparkinsonian medication. After informed consent was obtained, a trained neurologist performed the Movement Disorder Society–Unified Parkinson’s Disease Rating Scale (MDS-UPDRS) assessment, followed by eye-tracker calibration and completion of PS and AS tasks. Finally, a standardized Slovak neuropsychological battery [21] was administered. The examination sequence, together with the duration framing, is illustrated in Fig. 1.

### Eye-tracking apparatus and protocol

Eye movements were recorded using a CE-marked Mobile Eyebrain Tracker (Mobile EBT^®^, e(ye)BRAIN, SuriCog; 300 Hz sampling rate, 0.25° precision), with data acquisition and analysis performed using EyeParadigm^®^ software (v1.18). Binocular recordings were obtained, and saccade parameters were averaged across eyes to increase measurement robustness. Participants were seated in a darkened room approximately 60 cm from a 21-inch monitor, with head position stabilized using a chin rest. Calibration of eye-to-screen gaze mapping with 13 fixation points was performed at the start of each session and repeated if accuracy of the measurement of degrees of visual angle exceeded 0.5°.

In the gap-prosaccade paradigm, participants generated reflexive saccades toward a lateral target following a 200 ms gap between central fixation offset and target onset. The task consisted of 36 trials (18 left, 18 right; randomized) preceded by 5 practice trials. Outcome measures were mean saccadic latency and the percentage of EP (90–140 ms). Reflexive inhibitory control was indexed by EP percentage, i.e. the percentage of PS with latency falling into the EP range defined above.

In the gap-antisaccade paradigm, participants generated voluntary saccades opposite the target location. Visual timing and trial structure were identical to the gap-prosaccade task. Outcome measures included AS latency (> 140 ms; correct trials only) and AS error percentage (PS toward the target). Voluntary inhibitory control was indexed by AS latency and error rate.

### Motor and neuropsychological assessment, and questionnaire-based evaluations

The assessment sequence and estimated durations are shown in Fig. 1. All assessments were conducted using standardized and validated Slovak versions of the respective tests and questionnaires. Participants completed questionnaires independently or with assistance when required.

Motor symptom severity was assessed using the MDS-UPDRS.

Neuropsychological evaluation was performed using a Slovak battery [21], including the Montreal Cognitive Assessment (MoCA) assessing the global cognitive status; the Frontal Assessment Battery (FAB), phonemic verbal fluency, and the Trail Making Test parts A and B (TMT-A, TMT-B), capturing inhibitory control, cognitive flexibility, and strategic retrieval. Phonemic verbal fluency was assessed using the letters K and P (one minute each).

Global executive functioning was indexed using a composite executive function score derived from the FAB, phonemic verbal fluency, and the TMT switching cost (B–A). Raw scores were z-standardized across the full sample (PD + HC), with TMT B–A scores sign-inverted so that lower values reflected better performance; the composite score was computed as the mean of standardized measures. Internal consistency was moderate (Cronbach’s α = 0.56), consistent with the literature aiming at values ~ 0.4–0.6. Too high a value would make the composite score overly redundant with the individual items, whereas too low a value would indicate that the items do not reliably measure the same construct, reducing the validity of the composite score. Composite scores are supported by prior work showing that they reduce task-specific noise and provide more stable estimates than single tests [22].

As for the questionnaires, ICBs were evaluated using the QUIP-RS, assessing symptom severity across behavioral domains over the preceding four weeks. Trait impulsivity was measured using the BIS-11, and health-related quality of life using the Parkinson’s Disease Questionnaire–8 (PDQ-8).

### Statistical analysis

All statistical analyses were conducted in R (RStudio version 2023.3.1.446). An a priori power analysis for correlation analyses was performed using the *pwr* package, indicating that a sample size of 28 participants is required to detect a large correlation coefficient (*r* = 0.50) with a two-tailed test at a significance level of α = 0.05 and 80% statistical power.

Given the modest sample size and anticipated deviations from normality, all statistical analyses were a priori selected to be nonparametric to ensure robust inference. Group differences between patients with PD and HC in eye-tracking and clinical measures were examined using Wilcoxon rank-sum tests, with effect sizes quantified as rank-biserial correlations (r). To control for multiple comparisons, Benjamini–Hochberg (BH) false discovery rate (FDR) correction was applied.

Associations between eye-tracking parameters (AS latency, PS latency, EP percentage, and AS error percentage) and clinical variables, including LEDD, QUIP-RS, PDQ-8 and BIS-11, were assessed using Spearman rank correlation analyses within the PD group, with FDR correction applied for multiple testing.

For selected associations, causal mediation analyses (CMA) were conducted using the *mediation package*. Mediation analyses were exploratory and intended to assess statistical interrelationships rather than causal mechanisms. Statistical mediation effects were estimated by fitting multiple linear regression models for the mediator and the outcome, followed by formal mediation testing. Statistical inference was based on nonparametric bootstrap resampling (5,000 iterations) with percentile-based confidence intervals, and indirect (average causal mediation effect), direct, and total effects were reported.

## Results

### Descriptive statistics and group characteristics

The study included 29 patients with PD (10 females, 19 males) and 20 HC (13 females, 7 males). PD and HC groups were comparable in demographic and cognitive characteristics. No significant group differences were observed in age (U = 145.0, *p* = 0.188), sex distribution (Fisher’s exact test, *p* = 1.000), years of education (U = 313, *p* = 0.626), or global cognitive performance as assessed by the Montreal Cognitive Assessment (MoCA; U = 310, *p* = 0.689).

**Table 1:** Demographic and clinical characteristics of the Parkinson’s disease cohort (*n* = 29)

Variable	Median	IQR	Min	Max
Age	63	8	39	74
Education years	14	5	12	19
MoCA	27	3	19	30
PHQ-9	5	5	0	16
BIS-11	49	22	26	88
EF (z-score)	0.1	0.9	-1.8	0.7
AS latency (ms)	341.5	92.8	222	504.5
PS latency (ms)	227.8	105.8	175	351.8
EP (%)	4.8	9	0	18.8
AS error (%)	30	20	0	80
LEDD levodopa (mg)	425	1050	0	1896
LEDD agonists (mg)	210	300	0	525
LEDD total (mg)	500	1000	210	2575
QUIP-RS	9	12	0	32
Disease duration (years)	5	9	0	12
MDS-UPDRS III	13.5	15	0	28

*AS,* antisaccade; *BIS-11,* Barratt Impulsiveness Scale; *EF,* executive function composite score; *EP,* express prosaccades; *GAD-7,* Generalized Anxiety Disorder 7-item Scale; *LEDD,* levodopa equivalent daily dose; *MDS-UPDRS,* Movement Disorder Society–Unified Parkinson’s Disease Rating Scale; *mg,* milligrams; *MoCA,* Montreal Cognitive Assessment; *ms,* milliseconds; *PD,* Parkinson’s disease; *PHQ-9,* Patient Health Questionnaire 9-item scale; *PS, *latency: prosaccade latency; *QUIP-RS,* Questionnaire for Impulsive-Compulsive Disorders in Parkinson’s Disease-Rating Scale

The PD cohort consisted of patients with mild disease severity (Hoehn and Yahr stages 1–2) who were assessed in the ON-medication state. Treatment regimens included levodopa monotherapy (*n* = 9), dopamine agonist monotherapy (*n* = 7), and combined levodopa and dopamine agonist therapy (*n* = 13). Detailed demographic, clinical, oculomotor, neuropsychological, and questionnaire-based characteristics are presented in Tables 1 and 2.

**Table 2:** Demographic and clinical characteristics of the healthy control cohort (*n* = 20)

Variable	Median	IQR	Min	Max
Age	63.5	7.25	57	69
Education years	17	5	12	21
MoCA	27.5	3	22	30
PHQ-9	3	3.25	0	9
BIS-11	57	9.5	49	67
EF (z-score)	0.2	1	-1.5	1.5
AS lat. (ms)	293.4	65.4	199.8	413.5
PS lat. (ms)	218.1	49.3	160.5	330.3
EP (%)	2	3.6	0	5.3
AS error (%)	19	20	6	49

*AS,* antisaccade; *AS error rate,* percentage of antisaccade directional errors; *AS lat.,* antisaccade latency; *BIS-11,* Barratt Impulsiveness Scale; *EF,* executive function composite score; *EP,* express prosaccades; *MoCA,* Montreal Cognitive Assessment; *ms,* milliseconds; *PS lat.,* prosaccadic latency

### Group differences in eye-tracking and clinical measures between Parkinson’s disease patients and healthy controls

Group comparisons between PD patients and HC revealed significant differences in selected eye-tracking measures (Supplementary Table S2). Specifically, PD patients showed longer AS latencies compared with HC (W = 164, *p* = 0.011, *r* = 0.37), which remained significant after FDR correction (adjusted *p* = 0.037). In addition, the EP percentage was significantly higher in the PD group than in HC (W = 137, *p* = 0.002, *r* = 0.45; adjusted *p* = 0.012).

No significant group differences were observed in PS latency, AS error percentage, or in clinical and questionnaire-based measures, including the executive function composite score and BIS-11. Effect sizes for non-significant comparisons were small to moderate (*r* ≤ 0.23). Detailed table and boxplots illustrating group differences are provided in Supplementary Material (Table S1 and Fig. S1).

### Associations of eye-tracking parameters with impulsive-compulsive behaviors, levodopa equivalent daily dose, and quality of life in the Parkinson’s disease group

Within the PD group, the EP percentage showed a strong positive association with s-ICBs severity as measured by the QUIP-RS (ρ = 0.64, BH-adjusted *p* = 0.002) and a moderate positive association with LEDD (ρ = 0.53, BH-adjusted *p* = 0.023). EP percentage was also moderately associated with PDQ-8 (ρ = 0.47, BH-adjusted *p* = 0.049). In addition, LEDD was strongly positively associated with QUIP-RS scores (ρ = 0.64, BH-adjusted *p* = 0.002). No other significant associations with clinical or questionnaire-based measures were observed after FDR correction.

In additional exploratory analyses, dopamine agonist-derived LEDD was not significantly associated with any eye-tracking or clinical measure (Supplementary Material, Table S2).

To account for the potential influence of dopamine agonist exposure, we conducted an exploratory sensitivity analysis using partial Spearman correlations with dopamine agonist use (yes/no) included as a covariate. The association between EP percentage and subclinical impulsive-compulsive symptom severity remained essentially unchanged after adjustment (ρ = 0.64, *p* < 0.001; partial ρ = 0.65, *p* < 0.001).

Spearman rank correlation coefficients and their significance levels are presented in Table 3. The corresponding uncorrected and Benjamini–Hochberg (BH)-corrected p values are provided in the Supplementary Material (Table S3), together with pairwise scatterplots illustrating the relationships among oculomotor, executive, and clinical measures (Fig. S2).

**Table 3:** Spearman rank correlation matrix of oculomotor, clinical, and questionnaire measures

	EF	AS lat.	PS lat.	EP	AS err.	PDQ-8	LEDD	BIS-11	QUIP-RS
EF	1	-0.223	-0.274	-0.118	-0.382	-0.214	-0.003	-0.261	-0.219
AS lat.	-0.223	1	0.669**	-0.477*	0.06	-0.075	-0.128	0.161	-0.178
PS lat.	-0.274	0.669***	1	-0.531*	0.17	-0.105	-0.099	0.199	-0.134
EP	-0.118	-0.477**	-0.531**	1	-0.018	0.473*	0.529*	0.214	0.641**
AS err.	-0.382*	0.06	0.17	-0.018	1	-0.134	-0.202	-0.082	-0.232
PDQ-8	-0.214	-0.075	-0.105	0.473*	-0.134	1	0.127	0.273	0.44
LEDD	-0.003	-0.128	-0.099	0.529**	-0.202	0.127	1	0.198	0.643**
BIS-11	-0.261	0.161	0.199	0.214	-0.082	0.273	0.198	1	0.352
QUIP-RS	-0.219	-0.178	-0.134	0.641***	-0.232	0.44*	0.643***	0.352	1

Uncorrected *p* values are shown in the lower-left triangle, whereas Benjamini–Hochberg (BH) false discovery rate–corrected *p* values are shown in the upper-right triangle of the matrix. Significance levels are indicated as follows: * *p* < 0.05, ** *p* < 0.01, *** *p* < 0.001, for both uncorrected and BH-corrected associations. *AS,* antisaccade; *AS err.,* antisaccade error rate (% directional errors); *AS lat.,* antisaccade latency; *BIS-11,*  Barratt Impulsiveness Scale; *EF,*  executive function composite score; *EP,* express prosaccades; *PS lat.,* prosaccade latency; *QUIP-RS,* Questionnaire for Impulsive–Compulsive Disorders in Parkinson’s Disease–Rating Scale; *LEDD,* levodopa equivalent daily dose; *PDQ-8,* Parkinson’s Disease Questionnaire–8

### Clinical covariates of associations between eye-tracking measures, dopaminergic medication, and impulsive–compulsive behaviors

After controlling for motor severity (MDS-UPDRS III), partial Spearman correlations revealed a significant positive association between EP percentage and QUIP-RS (ρ = 0.57, BH-adjusted *p* = 0.003). EP percentage was also positively associated with LEDD (ρ = 0.60, BH-adjusted *p* = 0.002) and PDQ-8 (ρ = 0.47, BH-adjusted *p* = 0.011).

### Impulsive–compulsive behaviors as a mediator between levodopa equivalent daily dose and express prosaccades

**Fig. 2:**
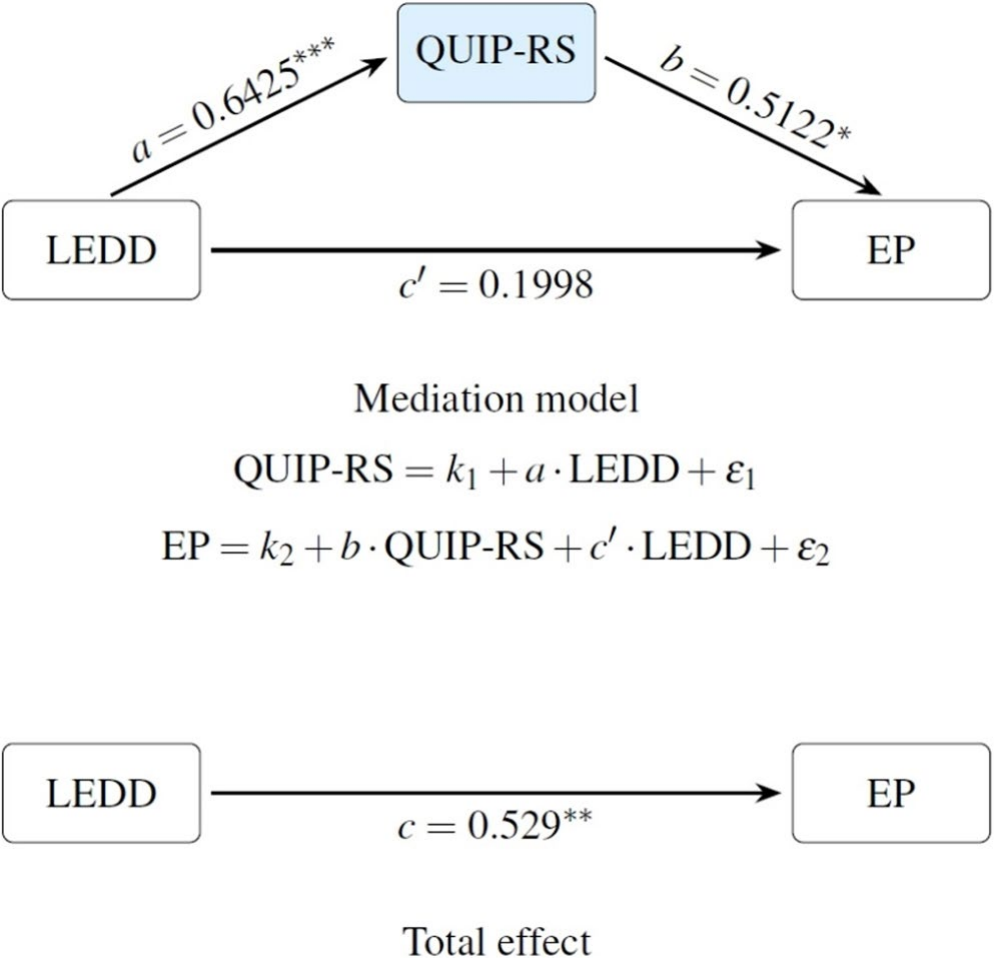
Mediation model of LEDD, QUIP-RS, and EP. Mediation analysis illustrating the indirect effect of LEDD on EP through QUIP-RS. Path *(a)* represents the association between LEDD and QUIP-RS, path *(b)* the association between QUIP-RS and EP controlling for LEDD, and path *(c′)* the direct effect of LEDD on EP after accounting for the mediator. The total effect of LEDD on EP *(c)* is shown in the lower panel. Coefficients are standardized regression estimates. Asterisks indicate statistical significance (* *p* < 0.05, ** *p* < 0.01, *** *p* < 0.001). EP = express prosaccades (%); LEDD = levodopa equivalent daily dose; QUIP-RS = Questionnaire for Impulsive–Compulsive Disorders in Parkinson’s Disease–Rating Scale. The schematic was created using LaTeX

Based on these associations, we exploratorily examined whether QUIP-RS scores were statistically consistent with an indirect association model linking LEDD and EP percentage (Fig. 2). Using z-standardized variables and nonparametric bootstrap resampling (5,000 iterations), the average causal mediation effect (ACME) was 0.329 (95% CI: 0.013–0.75, *p* = 0.042). The total effect of LEDD on EP percentage was 0.529 (95% CI: 0.274–0.73, *p* < 0.001). After inclusion of QUIP-RS in the model, the estimated direct effect was not statistically significant. The indirect pathway accounted for 62% of the total effect (95% CI: 0.019–1.80, *p* = 0.042).

## Discussion

This study examined the relationship between reflexive and voluntary oculomotor control, s-ICBs, trait impulsivity, and executive functioning in dopaminergically treated PD patients without clinically manifest ICDs. Three main findings emerged:

(i) Patients with PD showed group-level impairments in inhibitory oculomotor control compared with HC, reflected by prolonged AS latencies and increased EP frequency.
(ii) Within the PD group, EP but not voluntary oculomotor measures were selectively associated with the severity of s-ICBs, dopaminergic medication dose, and quality of life, indicating a specific link between reflexive inhibitory control and behavioral vulnerability in PD.
(iii) Mediation analyses indicated that the association between LEDD and EP was largely explained by s-ICBs severity, providing contextual support for the close interrelationship between dopaminergic treatment, behavioral symptoms, and reflexive oculomotor disinhibition.

At the group level, PD patients showed alterations in both rapid, automatic inhibitory processes and slower, voluntary control compared with HC. Longer AS latencies indicate less efficient top-down inhibitory control, whereas selectively elevated EP reflect reduced suppression of reflexive, stimulus-driven saccades. Importantly, mean PS latency did not differ between groups and was not associated with s-ICBs or motor severity. The preservation of PS latency, together with altered AS performance and increased EP frequency, therefore supports selective impairments in inhibitory control mechanisms rather than global motor impairment. This is consistent with prior evidence for abnormalities in both early and late inhibitory control in PD [9]. Global executive functioning and trait impulsivity did not differ significantly between groups.

Together, these findings indicate that oculomotor paradigms may capture subtle inhibitory abnormalities, particularly those related to s-ICBs, that are not reflected in standard clinical or neuropsychological assessments.

Within the PD group, reflexive oculomotor responses indexed by EP, but not voluntary measures, were selectively associated with s-ICBs, LEDD, and quality of life. In contrast, EP were unrelated to trait impulsivity as measured by BIS-11 and to the composite executive function score, indicating that EP do not simply reflect generalized impulsivity or executive dysfunction. This pattern is consistent with the computational model of inhibitory control in frontal cortex and basal ganglia [23], emphasizing the relevance of distinguishing inhibitory processes based on temporal dynamics. Importantly, all associations remained significant after controlling for motor severity, indicating that the relationships between EP, s-ICBs, medication dose, and quality of life are not driven by general motor impairment. This specificity of reflexive oculomotor control in relation to ICBs in PD is consistent with findings of Riek et al. showing that EP primarily load on a latent global inhibitory control factor, interpreted as impulsivity, rather than on voluntary or executive oculomotor control [24]. Together, these results support the specificity of reflexive oculomotor inhibitory control as a correlate of s-ICBs in PD.

Exploratory mediation analyses further indicated that the association between LEDD and EP was largely accounted for by the severity of s-ICBs, suggesting a close statistical interrelationship between dopaminergic treatment, behavioral symptoms, and reflexive oculomotor disinhibition rather than a direct effect of medication on motor output.

One possible mechanistic interpretation of these interactions is that EP may reflect alterations in fast, reflexive inhibitory gating within basal ganglia–superior colliculus (SC) circuitry. Rapid suppression of stimulus-driven saccades generally depends on phasic inhibitory signaling mediated by the hyperdirect cortico–subthalamic nucleus (STN)–substantia nigra pars reticulata pathway. In PD, this inhibitory influence may be less efficient or more variable, potentially resulting in a “leaky” inhibitory gate that allows some visually triggered saccades to escape suppression before slower, top-down control processes can engage [25]. Dopaminergic modulation of STN dynamics and changes in cortico–subthalamic coupling may further shape this process, possibly facilitating faster reflexive responses while sparing, or even improving, slower voluntary saccades [15, 26].

Although elevated EP rates are interpreted here as reflecting impaired reflexive inhibitory control, alternative mechanisms should be considered. In particular, dopamine can directly modulate the excitability of the SC through local receptor-mediated effects, in addition to its indirect influence via basal ganglia inhibitory circuits [27]. Another proposed mechanism is the dopamine overdose hypothesis, which proposes excessive dopaminergic stimulation of relatively spared striatal regions, potentially promoting impulsivity and reduced suppression of reflexive saccades [14]. However, several aspects of the present findings are less consistent with a dominant role of generalized motor facilitation or striatal overdose effects [28]. Voluntary oculomotor measures were not associated with LEDD, EP were unrelated to motor severity. In addition, effects predicted by overdose models [14] such as stronger disinhibition in patients with lower motor severity, were not observed. Finally, the mediation results and the absence of LEDD effects on overall PS latency may be more consistent with phasic failures of rapid inhibitory gating than with tonic dopaminergic effect producing generalized motor facilitation.

Somewhat surprisingly, no significant association was observed between dopamine agonist dose and oculomotor or behavioral measures in exploratory analyses, despite the well-established link between dopamine agonists and clinically manifest impulse control disorders. This null finding may reflect the modest sample size and limited statistical power to detect medication-class–specific effects, as well as the focus on subclinical impulsive–compulsive behaviors rather than overt ICDs. Furthermore, the heterogeneous dopaminergic treatment regimens and assessment exclusively in the ON-medication state may have reduced sensitivity to medication-specific effects. In addition, s-ICBs may arise from different or more distributed dopaminergic mechanisms than clinically manifest ICDs, and their expression may be influenced by cumulative dopaminergic exposure rather than dopamine agonist dose alone. Notably, impulsive–compulsive behaviors have also been reported in patients treated with levodopa monotherapy [29], indicating that the present findings are not necessarily inconsistent with existing literature.

The timing of testing may also be relevant. All assessments were performed approximately 1 h after the morning medication dose to standardize evaluation in the ON state. However, levodopa and dopamine agonists have markedly different pharmacokinetic properties, which may influence the pattern of dopaminergic stimulation at the time of assessment [30]. Although we did not observe significant effects of dopamine agonist exposure, the exploratory measures used in the present study may not have fully captured differences in dopamine agonist subtype, dosage, or duration of use.

Finally, moderate associations between EP, PDQ-8, and QUIP-RS indicate that s-ICBs can compromise quality of life even before reaching clinical diagnostic thresholds, highlighting the clinical relevance of the present study.

## Limitations and future directions

Several limitations should be acknowledged. We recognize the following three major ones:

(i) The cross-sectional design, modest sample size, and exclusive ON-medication assessments together limit causal inference, reduce statistical power, and prevent evaluation of medication-specific effects.
(ii) All assessments were conducted with the QUIP-RS, which is designed solely for patient populations, preventing direct comparison of s-ICBs between PD patients and HC.
(iii) The study did not include longitudinal measures, limiting the predictive value of oculomotor measures for the emergence of ICDs.

We propose several directions for future research. Comparing ON and OFF medication states would help clarify medication-specific effects on reflexive and voluntary inhibitory control. Incorporating additional task-based measures of inhibitory control, such as the Simon task, would allow assessment of convergent validity with oculomotor indices. Longitudinal or prospective designs could examine the development of s-ICBs over time and evaluate the predictive potential of oculomotor markers for the emergence of ICDs. In addition, statistical and machine learning approaches could be used for data mining and pattern recognition in oculomotor and clinical measures. This would allow identification of complex patterns in the data and enable models to predict the emergence of ICDs, as well as to evaluate whether subclinical impulsivity constitutes a risk factor for their development.

Some limitations, such as the inability to directly compare s-ICBs between PD patients and HC due to QUIP-RS constraints, cannot be fully resolved but could be mitigated by alternative assessment tools.

## Conclusion

In conclusion, reflexive, but not voluntary, oculomotor inhibitory control was selectively associated with s-ICBs, LEDD, and quality of life in PD. Exploratory mediation analyses suggest that dopaminergic medication may influence reflexive oculomotor disinhibition indirectly through behavioral changes rather than pure motor facilitation.

This pattern highlights the potential specificity of reflexive oculomotor processes and underscores the importance of distinguishing reflexive from voluntary inhibitory mechanisms for understanding non-motor manifestations of the PD. Future longitudinal studies are needed to clarify the clinical relevance and predictive value of these reflexive oculomotor alterations.

## Supplementary Information

Below is the link to the electronic supplementary material.

Supplementary Material 1

## Data Availability

The datasets generated during and analysed during the current study are available from the corresponding author on reasonable request.
