# Supplementary material for: Reflexive eye movement alterations are related to subclinical impulsive-compulsive behaviors in Parkinson’s disease
Source: Neurol Sci. 2026 Jun 19;47(7):578. doi: 10.1007/s10072-026-09177-z (PMC13282338; doi:10.1007/s10072-026-09177-z)
Supplement: Supplementary file 1 — Supplementary Material 1 [file 10072_2026_9177_MOESM1_ESM.docx]

**Supplementary Material**

**Article title**

Reflexive Eye Movement Alterations Are Related to Subclinical Impulsive–Compulsive Behaviors in Parkinson’s Disease

**Journal**

Neurological Sciences

**Authors**

Lenka Hapakova^a^, Igor Straka^b^, Jan Necpal^a,b^, Alice Kusnirova^a^, Pavol Martis^a^, Peter Valkovic^a,c^, Zuzana Kosutzka^a^

**Affiliations**

1. 2nd Department of Neurology, Comenius University Faculty of Medicine, University Hospital Bratislava, Limbova 5, 833 05 Bratislava, Slovakia
2. Neurology Department, Hospital Zvolen, a. s., Kuzmanyho nabrezie 28, 960 01 Zvolen, Slovakia
3. Centre of Experimental Medicine of the Slovak Academy of Sciences, Institute of Normal and Pathological Physiology, Dubravska cesta 9, 841 04 Bratislava, Slovak Republic

**Corresponding author**

Zuzana Kosutzka, 2nd Department of Neurology, Comenius University Faculty of Medicine, University Hospital Bratislava, Slovakia, e-mail: zuzanakosutzka@gmail.com

|  | | | | |
| --- | --- | --- | --- | --- |
|  |  |  |  |  |
|  |  |  |  |  |
|  |  |  |  |  |
|  |  |  |  |  |
|  |  |  |  |  |
|  |  |  |  |  |
|  |  |  |  |  |
|  |  |  |  |  |
|  |  |  |  |  |
|  |  |  |  |  |
|  |  |  |  |  |
|  |  |  |  |  |
|  |  |  |  |  |
|  |  |  |  |  |
|  |  |  |  |  |
|  |  |  |  |  |
|  |  |  |  |  |
|  | | | | |

|  | | | | |
| --- | --- | --- | --- | --- |
|  |  |  |  |  |
|  |  |  |  |  |
|  |  |  |  |  |
|  |  |  |  |  |
|  |  |  |  |  |
|  |  |  |  |  |
|  |  |  |  |  |
|  |  |  |  |  |
|  |  |  |  |  |
|  |  |  |  |  |
|  |  |  |  |  |
|  | | | | |

| **Table S1** Group differences between Parkinson’s disease patients (n = 29) and healthy controls (n = 20) | | | | | |
| --- | --- | --- | --- | --- | --- |
| Variable |  | W | p | Effect size r | p adj. |
| EF |  | 314 | 0.633 | 0.068 | 0.633 |
| AS lat. |  | 164 | 0.011 | 0.365 | 0.037* |
| PS lat. |  | 240 | 0.314 | 0.144 | 0.314 |
| EP |  | 137 | 0.002 | 0.449 | 0.012* |
| AS err. |  | 211 | 0.110 | 0.228 | 0.154 |
| BIS-11 |  | 337.5 | 0.34 | 0.136 | 0.386 |
| Effect size is reported as rank-biserial correlation (r). Adjusted p values reflect Benjamini–Hochberg (BH) false discovery rate correction. *p < 0.05 after BH correction. AS = antisaccade; AS err. = antisaccade error rate (% directional errors); AS lat. = antisaccade latency; BIS-11 = Barratt Impulsiveness Scale; EF = executive function composite score; EP = express prosaccades; PHQ-9 = Patient Health Questionnaire–9; PS lat. = prosaccade latency | | | | | |


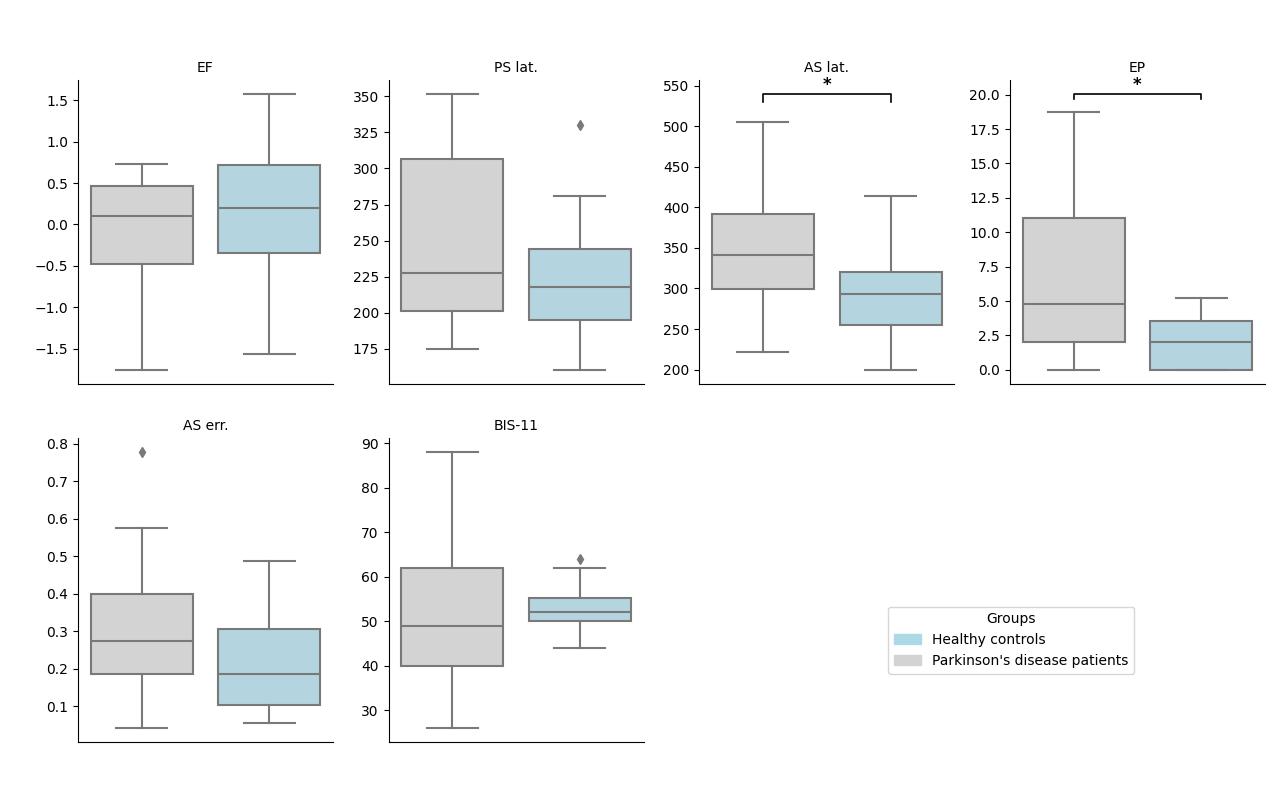
**Fig. S1** Group differences in eye-tracking and clinical measures between Parkinson’s disease patients and healthy controls. Blue boxes represent healthy controls; gray boxes represent Parkinson’s disease patients. AS = antisaccade; AS err. = antisaccade error rate (% directional errors); AS lat. = antisaccade latency; BIS-11 = Barratt Impulsiveness Scale; EF = executive function composite score; EP = express prosaccades; PS lat. = prosaccade latency. *p < 0.05 after Benjamini–Hochberg correction

**Table S2** Spearman correlations between dopamine agonist LEDD (mg) and oculomotor measures

| **Measure** | **ρ** | **p-value** | **p (BH)** |
| --- | --- | --- | --- |
| **EP (%)** | 0.159 | 0.334 | 0.706 |
| **PS lat. (ms)** | 0.022 | 0.895 | 0.895 |
| **AS lat. (ms)** | 0.041 | 0.805 | 0.895 |
| **AS error (%)** | -0.123 | 0.457 | 0.706 |

Spearman’s rank correlation coefficients (ρ) are reported together with uncorrected p values and Benjamini–Hochberg (BH) false discovery rate–corrected p values. EP = express prosaccades (%); PS lat. = prosaccade latency (ms); AS lat. = antisaccade latency (ms); AS error = antisaccade error rate (% directional errors); LEDD = levodopa equivalent daily dose (mg).

| **Table S3** Matrix of p values for Spearman rank correlations among oculomotor, clinical, and questionnaire measures | | | | | | | | | |
| --- | --- | --- | --- | --- | --- | --- | --- | --- | --- |
|  | **EF** | **AS lat.** | **PS lat.** | **EP** | **AS err.** | **PDQ-8** | **LEDD** | **BIS** | **QUIP-RS** |
| **EF** | 0 | 0.521 | 0.457 | 0.675 | 0.164 | 0.521 | 0.989 | 0.474 | 0.521 |
| **AS lat.** | 0.245 | 0 | 0.002 | 0.049 | 0.803 | 0.761 | 0.657 | 0.608 | 0.58 |
| **PS lat.** | 0.15 | 0 | 0 | 0.023 | 0.593 | 0.706 | 0.708 | 0.521 | 0.657 |
| **EP** | 0.543 | 0.009 | 0.003 | 0 | 0.952 | 0.049 | 0.023 | 0.521 | 0.002 |
| **AS err.** | 0.041 | 0.758 | 0.379 | 0.925 | 0 | 0.657 | 0.521 | 0.756 | 0.521 |
| **PDQ-8** | 0.266 | 0.698 | 0.588 | 0.01 | 0.488 | 0 | 0.657 | 0.457 | 0.077 |
| **LEDD** | 0.989 | 0.51 | 0.61 | 0.003 | 0.294 | 0.511 | 0 | 0.521 | 0.002 |
| **BIS** | 0.171 | 0.405 | 0.301 | 0.266 | 0.672 | 0.152 | 0.304 | 0 | 0.219 |
| **QUIP-RS** | 0.254 | 0.355 | 0.487 | 0 | 0.225 | 0.017 | 0 | 0.061 | 0 |
| Uncorrected p values are shown in the lower-left triangle, whereas Benjamini–Hochberg (BH) false discovery rate–corrected p values are shown in the upper-right triangle of the matrix. Significance levels are indicated as follows: * p < 0.05, ** p < 0.01, *** p < 0.001, for both uncorrected and BH-corrected associations. AS = antisaccade; AS err. = antisaccade error rate (% directional errors); AS lat. = antisaccade latency; BIS-11 = Barratt Impulsiveness Scale; EF = executive function composite score; EP = express prosaccades; PS lat. = prosaccade latency; QUIP-RS = Questionnaire for Impulsive–Compulsive Disorders in Parkinson’s Disease–Rating Scale; LEDD = levodopa equivalent daily dose; PDQ-8 = Parkinson’s Disease Questionnaire–8 | | | | | | | | | |


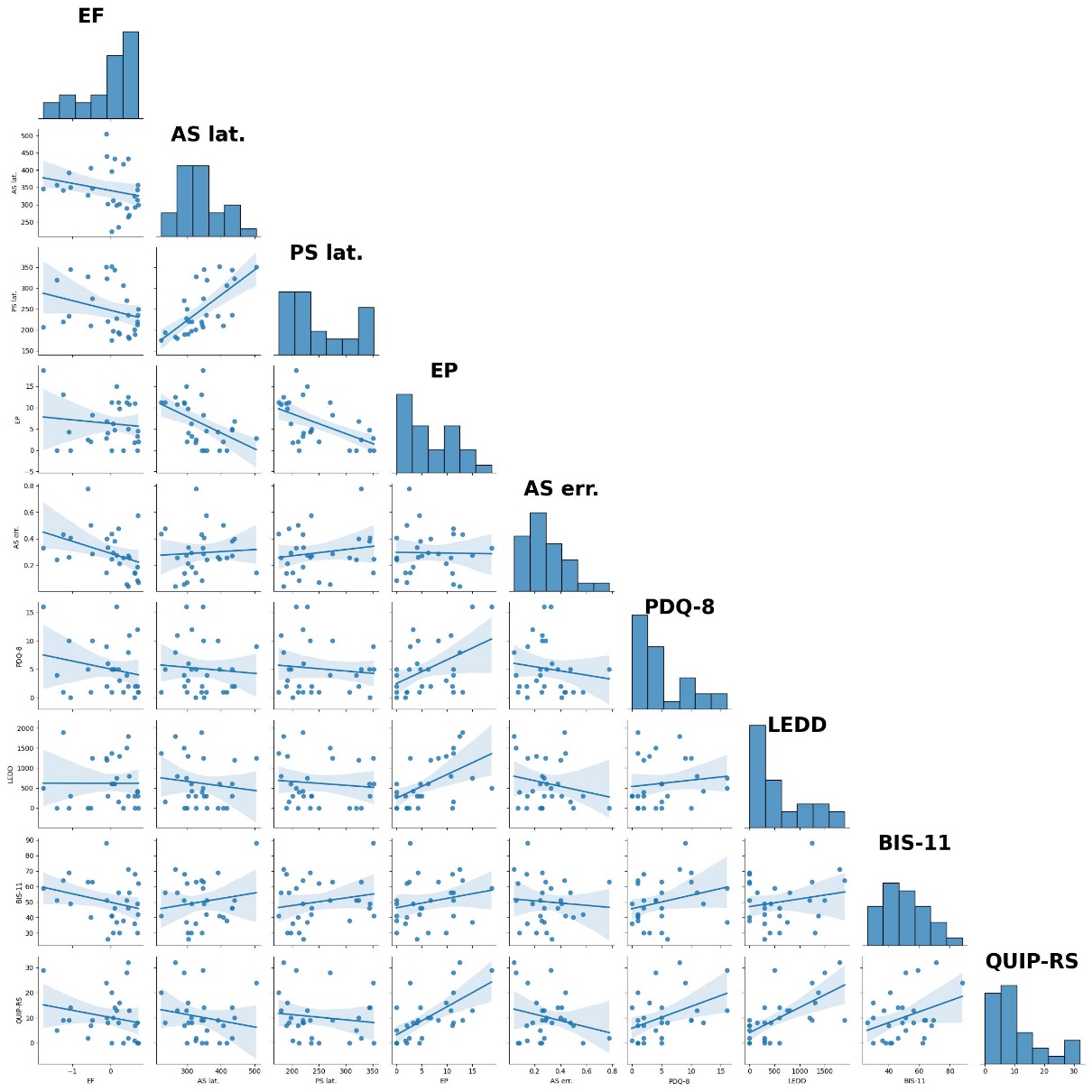


**Fig. S2** Scatterplot matrix illustrating relationships between oculomotor performance, executive function, and clinical measures in Parkinson’s disease. Panels show pairwise scatterplots with fitted linear regression lines and 95% confidence intervals; histograms on the diagonal depict variable distributions. EF = executive function composite score; AS lat. = antisaccade latency; PS lat. = prosaccade latency; EP = express prosaccades; AS err. = antisaccade error rate (% directional errors); PDQ-8 = Parkinson’s Disease Questionnaire–8; LEDD = levodopa equivalent daily dose; BIS-11 = Barratt Impulsiveness Scale; QUIP-RS = Questionnaire for Impulsive-Compulsive Disorders in Parkinson’s Disease–Rating Scale
